# How should teachers tackle students' boredom in the emergency online language class?

**DOI:** 10.3389/fpsyg.2022.1031515

**Published:** 2022-12-08

**Authors:** Qiong Huang, Xinmin Zheng

**Affiliations:** ^1^School of Chinese Studies and Exchange, Shanghai International Studies University, Shanghai, China; ^2^School of Education, Shanghai International Studies University, Shanghai, China

**Keywords:** boredom, COVID-19 pandemic, online setting, language learning, ecological perspective, nested ecosystems model

## Introduction

Owing to the outbreak of COVID-19 pandemic in 2020, the global education environment has experienced an abrupt and unprecedented conversion from the traditional face-to-face offline class to an underprepared computer-assisted online one (UNESCO, 2020; Limniou et al., 2021; Pokhrel and Chhetri, 2021; Pressley and Ha, 2021; Chen et al., 2022). Not only education authorities and schools of all levels, but also teachers, students as well as parents need to immediately face such a rapid shift (Kim and Asbury, 2020; Tadesse and Muluye, 2020; Gao et al., 2021; Vijayan, 2021; Ashton, 2022). Emergency remote teaching has thus become a necessity in such a pandemic age. Consequently, the modes of teaching and learning have been greatly changed, with teachers teaching on this side and students learning on the other side in those most risky places in the world (Luo et al., 2020; UNESCO, 2020; Chiu Thomas et al., 2021; Tsang Jenny et al., 2021; Kupers et al., 2022). Boredom, a term describing students' learning behaviors, captured the attention of certain Second Language Acquisition researchers under such circumstances (see Derakhshan et al., 2021, 2022; Yazdanmehr et al., 2021; Kruk et al., 2022; Li, 2022), though grassroots teachers might ignore this negative emotion from their students' struggles (Derakhshan et al., 2021). As quite a few research studies in the past decade (see Pekrun et al., 2010; Chapman, 2013; Kruk and Zawodniak, 2018, 2020; Pawlak et al., 2020, 2022; Li, 2021) have shown that boredom can hinder learning performance and even result in poorer achievement outcomes, it deserves further investigations and deeper insights into the causal mechanisms of boredom emergence, especially in the terrain of emergency remote language classes in the current situation, which is most probably going to stay for long (Yazdanmehr et al., 2021; Derakhshan et al., 2022).

With concerns for students' boredom in our own emergency remote teaching and academic interest *per se*, we read the newly-published article by Kruk et al. (2022), which intended to probe into the ecosystemic factors underneath the emergence of boredom in an online English language classroom. To be more specific, the article took an ecological perspective to explore the causes of boredom experienced online by four 14-year-old EFL learners affected by different ecosystemic elements *via* the nested ecosystems model during the pandemic. In our view, the article reached a conclusion filled with both interpretative and illuminating evidence which is directive in coping with student boredom online for both practitioners and researchers in different ecosystemic layers. Therefore, we would like to share our opinions with our readers, hoping to provide heuristic comments for further investigation and more rational online practice. To achieve clarity, the article Kruk et al. (2022) will be referred to as “the article” hereby.

Prior to our detailed analysis of the article, we believe it is necessary to make clear the existing causes of boredom used in the article, together with how it can be embedded in the nested ecosystems model. Admittedly, the emergence of boredom can be ascribed to multifarious factors based on different models and theories (see Hill and Perkins, 1985; Larson and Richards, 1991; Pekrun, 2006; Eastwood et al., 2007, 2012; Pekrun et al., 2010; Davies and Fortney, 2012; Tulis and Fulmer, 2013). To sum up as a whole, the absence of challenging stimuli, teachers' excessive control and monotonous talk, students' limited choice, students' poor self-regulate attention and self-awareness, students' low perception of task control and value, students' own emotion identification as well as the over- or under-consumed mental energy, are the main sources of boredom emergence according to the abovementioned works. As for the online language learning context, Derakhshan et al. (2021) uncovered teachers' long, tedious monologs, students' insufficient participation, carelessly-chosen tasks and technical problems as the main sources of boredom, while Yazdanmehr et al. (2021) revealed user-unfriendly requirements of online education, such as physical distance or sedentary position, are antecedents of boredom likewise. Moreover, Dewaele et al. (2022) found that the lack of live personal interactions and the monotonous teacher delivery could induce students' disengagement and cause boredom.

Based on the identified causes of boredom in language learning, the article proposed a perspective of ecology to examine the emergence of boredom in an emergency remote English class influenced by different ecosystemic factors. The nested ecosystems model of ecological models serves to explore human behaviors from the immediate to the interdependent and overarching social cultural context (Bronfenbrenner, 1979, 1993), so individual students involved in the online setting of an EFL course can be embedded in different ecosystemic layers to gain a distinctive insight into their perceptions and interpretation of boredom. The article linked microsystem, the inner-most layer of the nested ecosystems model to the online classroom setting, where teachers, students, tasks and activities interacted; mesosystem to students' past online language learning experiences with the current online setting; exosystem to the school policies on online English study; and macrosystem, the outmost of the model, to cultural beliefs and public views of students' emergence of boredom online.

## The study

Guided by the sole research question “What are the ecosystemic factors underlying the emergence of boredom in an online English language classroom?” (Kruk et al., 2022), the article adopted a case study with four participants to analyze and compare.

The four participants (one male and three females) were from a private English institute in Iran and were selected *via* a deviant case sampling strategy. A questionnaire was used to decide the subsequent four interviewees, and then two interviews were carried out with a 2-week interval in between to get the qualitative data. After that, the interviews were transcribed and translated into English, followed by reading, coding, categorizing and theorizing under the different ecosystemic layers. It is worth mentioning that there is a complete questionnaire and interview questions attached to the article as appendixes, as well as how the interview questions were designed in line with the ecosystemic framework. Both of the two research tools may facilitate and give reference for future related researches.

In line with the acquired sources of student boredom in either online or offline learning contexts and the four layers of nested ecosystems model, the article illuminated the interaction between ecosystemic factors and participants' boredom in the online English class from the qualitative data through the interviews. At the microsystem level, the teacher's lengthy monologs and repetitive activities under or above students' competence failed to arouse participants' attention for long and resulted in their feeling valueless or exhausted in doing the tasks. To ensure online classroom discipline, the teacher had to exert greater control by muting their microphones, leaving the participants' boredom getting intensified before being given opportunities to talk. At the mesosystem level, home distraction appeared to be the culprit where noise disturbance easily took away the participants' attention or interrupted their tasks underway, and thus a lost track of the class triggered further boredom. Meanwhile, the participants' previous dissatisfied online learning experiences overshadowed their current online learning engagement, especially when the teacher followed the same instruction route as usual. At the exosystem level, the participants' physical fatigue from the long class period, intensive workload with short session intervals but unchallenging online assessment, as well as frequent changes of online platforms by the institute, intertwined to their boredom arousal. As Van Lier (2004) claimed, ecology is the study of one organism holds with the others and with the environment. Although the three ecosystemic levels entail their specific boredom factors corresponding to those theories and models of boredom, several factors embedded in one layer can influence the other layer factors in the emergence of boredom, aligned or misaligned (Kruk et al., 2022), for example, the conflict between home distraction in the mesosystem and the participants' adaption to the online setting after overcoming boredom for a period in the microsystem. As we see it, several factors in the emergence of boredom overlap and interact within the ecosystem.

Significantly, by taking the ecological perspective, the article echoed the call for situating the emergence of student boredom within the ecology of online classroom learning (Yazdanmehr et al., 2021), and thus put forward a thought-provoking framework for investigating further reasons and indicating solutions for students' diminished interest and participation under the interplay of each ecosystemic layer.

What' s more, the research into boredom in an online language class setting adds to the scarcity of studies on sources of boredom in emergency remote teaching during the seemingly endless COVID-19 pandemic. In this sense, we deem that the article is of strong timeliness and of reference value for analogous studies in other specific online contexts of language learning universally and help to raise the quality of online education by understanding more about this negative emotion.

## Discussion

Undoubtedly, the article provides an exemplary case study with abundant evidences for understanding student boredom in the online setting from an ecological perspective, shedding light on how this negative emotion is linked to the environment beyond the classroom. It is of great value in making up for research insufficiency while inspiring researches to dig into the combination of emotions and contextual factors. Nevertheless, in this part, we want to make our comments on the research design, theme creating and other points of the article in the hope of facilitating further research.

Firstly, a questionnaire was used to select the participants who were of the highest or the lowest scores. To make the selection more persuasive, an experienced teacher of this class was involved in the process, but it remains unknown how she finally helped determine the four cases. As Elman and Kapiszewski (2014) indicate that researchers are supposed to share the evidentiary basis and explain how the conclusions are reached. Then, in the study, two semi-structured interviews were the only sources to collect interpretive data from the participants, which seemed to be too one-sided. To ensure the depth and width of qualitative data as far as possible, several sources of data, need to be combined to reach triangulation (Creswell, 2014; Rossman and Rallis, 2016). Hence, it is obvious that more data collection sources can be introduced to consolidate the reliability and validity (Creswell, 2007) of the influential factors of students' boredom in the article. For instance, online class observation would be appreciated in unveiling the real conditions of student learning and teacher teaching. Qualitative studies using field or classroom observations (Tao and Gao, 2017; Ruan and Zheng, 2019; Mansouri et al., 2021; Zhang et al., 2022) have well illustrated that it is an effective way to understand the participants' real practice and complement the data that may have been missed in the interviews. Besides, data from teachers, parents as well as school administrators can be added to supplement to students' unilateral comments or complaints. At last, as addressed by the researchers themselves, further ecological studies could be conducted in other contexts to gain the vacant information hereby concerning the macrosystem factors impacting students' boredom experience. All in all, there is still space for researchers to enhance in terms of data collection in future related studies.

Secondly, to identify themes and sub-themes from the qualitative data through interviews with four participants, the article conducted the practice based on Bronfenbrenner's (1979) nested ecosystems model. Key concepts were picked out to form sub-themes or codes and then themes came into being. As is known to us, the analytic goals of theming are to winnow down the number of themes to explore and to develop an overarching theme from the data corpus (Saldaña, 2016) and the winnowing down of themes are supposed to be “essential” rather than “incidental” (van Manen, 1990). However, some themes in the microsystem appeared to be somewhat repetitive. In the article, the teachers' lengthy, repetitive talk reflected the boring and teacher-centered “teaching style,” whereas the “lack of peer participation and engagement” stemmed from the teacher's lengthy talk actually mirrored the fact that the class was never student-centered, which may be classified into “teacher's teaching style,” too. In addition, the theme “class control” manifested the teacher had to control students' by muting their microphones due to possible class chaos, whereas “students' violation of class discipline” also dealt with students' annoying behaviors online. Similarly, repetitive themes can also be found in the exosystem. The two themes “online platform problems” and “low learners' literacy of using the online platform” are both about boredom from online platform problems, including frequent changes of platforms and students' low literacy in operating different platforms, so they may as well be classified together likewise. In effect, Jeon et al. (2022) have pointed out that all codes that share a similar core meaning are likely to be grouped into a common theme through several rounds of discussions.

Thirdly, sources of students' boredom in the online setting revealed by the article are distinct from that of the traditional face-to-face class, which may be suggestive for teachers and administrators to make adjustments in different ecosystemic layers. However, the article appeared to be a bit absolute by addressing that it will be easier to vary the teaching style in offline instruction (see Kruk et al., 2022, p. 12). According to one of the participants' remark (see Kruk et al., 2022, p. 7), the teacher's online teaching style is almost a duplication of what she did offline, indicating there being no change in two different teaching settings. Interestingly, though, it brings about other potential fields worth investigating between teacher-student classroom practice, their emotions and teaching-learning outcomes either online or offline. On one hand, as teachers are supposed to respond and make changes to maintain student learning and to innovate or make changes (Hitlin and Elder, 2007; Ehren et al., 2021), teacher efficacy, teacher agency or digital affordances, etc. in front of such emergency remote teaching under COVID-19 should all be taken into consideration. Actually, teacher efficacy of virtual instruction and engagement were found to be negatively affected during the pandemic in 2020 (Pressley and Ha, 2021), causing teacher stress or even burnout. On the other hand, students' self-efficacy, academic emotions, EFL proficiency (Wang et al., 2021) or learning motivation and learning strategies (Chiu Thomas et al., 2021; Randi and Corno, 2022) are also intertwined with teachers' teaching practice, generating reciprocal impact on teaching quality. As far as we are concerned, the variables mentioned above concerning teachers and students as a whole should be good entries to investigate students' boredom in remote teaching and learning in the future.

## Conclusion

In summary, the article is a well-explored and far-reaching piece. It reveals that the online classroom is never an isolated virtual world and the emergence of student boredom is triggered by different factors in the ecosystem as a whole, which either encompass or interact with each other. We are convinced that, after reading the article, researchers, practitioners, school authorities and even parents can get a better understanding into what underlies students' boredom in remote language classes so as to tackle the problem appropriately in future studies, teaching practices, course designs and family environment building. Therefore, we feel it meaningful to recommend the article to more readers, particularly those who are in the pursuit of improving teaching quality and students' sustainable learning engagement in the online setting or in other contexts.

## Author contributions

QH and XZ selected the commented article together. QH drafted the opinion. XZ provided insights and valuable suggestions during her writing and helped revise the text. Both authors contributed to the article and approved the submitted version.
